# The Israeli Face Database (IFD): A multi-ethnic database of faces with supporting social norming data

**DOI:** 10.3758/s13428-025-02723-1

**Published:** 2025-06-13

**Authors:** Maayan Trzewik, Mayan Navon, Tal Moran, Hadas Wardi, Adi Langer, Bat-Sheva Hadad, Carmel Sofer, Niv Reggev

**Affiliations:** 1 Department of Psychology, Ben-Gurion University of the Negev, Beer Sheva, Israel; 2 Department of Education and Psychology, The Open University of Israel, Ra’anana, Israel; 3 Psychological Sciences Research Institute, UCLouvain, Ottignies-Louvain-la-Neuve, Belgium; 4 Department of Special Education, University of Haifa, Haifa, Israel; 5 Department of Brain and Cognitive Sciences, Ben-Gurion University of the Negev, Beer Sheva, Israel; 6 Department of Industrial Engineering and Management, Ben-Gurion University of the Negev, Beer Sheva, Israel; 7 School of Brain Sciences and Cognition, Ben-Gurion University of the Negev, Beer Sheva, Israel

**Keywords:** Face database, Face perception, Emotional expression, Social perception, Diversity, Israel

## Abstract

Human faces and their representations play a key role in social interactions. Consequently, face images are widely used as stimuli across psychological research. However, most available face databases primarily represent Western, predominantly White populations, raising concerns about the generalizability of findings derived from such nondiverse stimuli sets. The current work addresses this problem by introducing the Israeli Face Database (IFD), a novel database presenting face images of real individuals from an ethnically diverse population underrepresented in psychological research on face perception—people residing in Israel. The IFD features multiple highly controlled images per face identity, presenting several facial expressions. Additionally, we provide detailed demographic data on the face identities, expression validation data rated by Israeli participants, and social norming data rated by both Israeli and US-based participants. Validation data (Study 1) confirmed that the expression images in the IFD accurately convey their intended expressions. Norming data analysis (Study 2) revealed that judgments of IFD faces were broadly consistent across cultures, with notable variations in perceived typicality and perceived ethnic diversity. These results highlight the IFD’s strong psychometric properties and its ability to capture nuanced cultural and individual differences in face perception. We propose that the IFD can serve as a valuable tool for diversifying stimuli in social-psychological research.

**Supplementary Information:**

The online version contains supplementary material available at 10.3758/s13428-025-02723-1.

The human face is a rich source of invaluable information for social interactions. The features present on or perceived from an individual’s face can provide key information about their identity, social group membership, emotional state in response to their surrounding environment, and potential intentions toward others (Ekman & Oster, 1979; Freeman et al., 2010a, 2010b; Freeman & Johnson, 2016; Hugenberg & Wilson, 2013; Kawakami et al., 2017; Matsumoto et al., 2008; Todorov et al., 2015; but see Aviezer et al., 2012; Hassin et al., 2013, for the influence of context on interpreting facial features). Thus, faces play a vital role in the complex dynamics of human social interaction and understanding. Moreover, impressions made based on facial features are formed rapidly (Ito & Urland, 2003, 2005; Oosterhof & Todorov, 2008; Willis & Todorov, 2006), even without much information (e.g., Ambady et al., 1999; Rule & Ambady, 2008, 2010). These impressions, in turn, have been shown to significantly influence the decisions and choices made by perceivers regarding the individuals they encounter (e.g., Ballew & Todorov, 2007; Correll et al., 2002; Todorov et al., 2005; Wilson & Rule, 2015; Wilson et al., 2017).

Given their profound importance to understanding human interaction, it is not surprising that faces are widely used as stimuli across fields of psychological research (for a review, see Strohminger et al., 2016). For example, facial stimuli are used to investigate questions about the emotions experienced by others (Adolphs, 2002; Ekman & Oster, 1979; Izard, 1990; Jack et al., 2016; Matsumoto et al., 2008), the way people form impressions of others (Jaeger & Jones, 2022; Oosterhof & Todorov, 2008; Todorov et al., 2005, 2008; Xie et al., 2021), how others are represented in people’s minds (Brinkman et al., 2017; Dotsch & Todorov, 2012; Jones et al., 2021; Ratner et al., 2014; Stolier et al., 2018), and what types of knowledge are triggered upon exposure to an individual’s face (Blair & Banaji, 1996; Chua & Freeman, 2022; Chwe & Freeman, 2024; Freeman et al., 2015, 2010a, 2010b; Ito & Urland, 2003, 2005; Kunda & Spencer, 2003; Stolier & Freeman, 2016). Facial stimuli are also used to investigate how people encode and process faces and their features. Researchers have explored questions such as how well people remember the faces of others (Bothwell et al., 1989; Shapiro & Penrod, 1986) and the reasons for difficulty in remembering the faces of people from other social groups (Hugenberg et al., 2010; Hugenberg & Sacco, 2008; Hughes et al., 2019; J. Lee & Penrod, 2022; Meissner & Brigham, 2001; Reggev et al., 2020; Schwartz et al., 2023; Stelter et al., 2023; Stelter & Degner, 2018; Trzewik et al., 2025; Valentine, 1991, 2005).

However, a significant limitation in most research using facial stimuli is the overreliance on participant populations and research materials originating from the United States and Western Europe (Henrich et al., 2010; Martinez, 2025; Rad et al., 2018). This reflects a broader issue in psychological science: the predominance of homogeneous, White, Western populations in both participants and materials (Henrich et al., 2010). Such reliance implicitly assumes that individuals across cultures and regions process and react to information in similar ways—an assumption consistently refuted across numerous mental faculties (e.g., Apicella et al., 2020; Blasi et al., 2022; Cheon et al., 2020; Henrich et al., 2010; Thalmayer et al., 2021). Ample research suggests that cultures and regions differ in how they process information from faces (e.g., Blais et al., 2021; Gendron et al., 2014; Jack et al., 2012; Martinez, 2022). Furthermore, reviews of the present state of the facial stimuli databases commonly used in psychological research have revealed that most databases consist mainly of faces of individuals from the United States and Western Europe. These databases often rely on participants from the United States as the participants that determine which attributes and characteristics are perceived from the faces (e.g., Bainbridge et al., 2012; Langner et al., 2010; Ma et al., 2015). This relatively narrow focus on a small subset of face ethnicities, gathered from participants residing in limited parts of the world, relies on limited cultural and historical contexts, thus casting doubt on the generalizability of the conclusions drawn from these data (Cikara et al., 2022; Shiramizu et al., 2024; Zivony et al., 2023). Unfortunately, thus far, efforts to expand the diversity of ethnicities of photographed faces have focused chiefly on ethnicities prominent in the US context (Chen et al., 2021; Conley et al., 2018; LoBue & Thrasher, 2015; Ma et al., 2015, 2021; Meyers et al., 2023; Pickron et al., 2024; Strohminger et al., 2016; Tottenham et al., 2009; Ueda et al., 2019; see Courset et al., 2018; Lakshmi et al., 2021; Sacco et al., 2016; Saribay et al., 2018, for exceptions). Valid inferences on human social face processing should not be limited to Western-based social face processing. Therefore, there is a need for increased global diversity in the human faces used in psychological research and the people who judge these faces.

## Possible solutions

Several methodological approaches could be used to create a face database representing human faces from traditionally underrepresented populations. These include the artificial generation of faces using morphing techniques, pure artificial intelligence models, the collection of real faces from online browsers and social media (i.e., face photographs collected “in the wild”), and the creation of new databases focusing specifically on underrepresented populations. In the following sections, we review the first three approaches, highlighting their benefits and limitations, and then introduce the final approach, which we adopted in the current study.

### Face morphs

One approach to generating faces of different ethnicities is using morphing techniques. Here, researchers select several real faces that serve as the “base” faces and combine them into a single face that is the average of the base faces (e.g., Byatt & Rhodes, 1998; Peery & Bodenhausen, 2008; Saribay et al., 2024). This approach, often used in studies of multiracial faces, allows the researcher to control the exact facial features believed to comprise the face of the ethnicity (or race) of interest (e.g., Krosch & Amodio, 2014; Pauker et al., 2009; Peery & Bodenhausen, 2008). However, despite the benefits of reasonable control of features, there are nevertheless some limitations to the use of such techniques in the attempt to create faces of different ethnicities. First, this technique is not equipped to generate faces of ethnicities that are not a combination of other, more common ethnicities (as in the case of multiracial people). For example, no combination of base faces could accurately create an Amahara person’s face. Moreover, as the morphing technique produces average faces, any unique expression or imperfection that could characterize a natural face would be smoothed out in the average face. Furthermore, and perhaps most importantly, generating morphs relies heavily on the assumptions and intuitions of the team that creates the morphs, thus potentially reifying stereotypical cultural perceptions that will manifest in the generated stimuli and their perceptions (Martinez, 2022). Indeed, studies comparing participants’ judgments of real biracial faces to those of morphed biracial faces have shown that the two are judged differently (Gaither et al., 2019; Ma et al., 2022). Thus, the averaged morphed face cannot serve as an ecological stimulus that resembles the actual faces of the ethnicity of interest.

### Artificially generated faces

Another approach to generating faces of underrepresented populations is using deep learning models (Elasri et al., 2022). These models enable researchers to synthetically generate face images that are difficult to distinguish from real faces that exist in the world (e.g., generative adversarial networks, Goodfellow et al., 2020; autoregressive models, Van den Oord et al., 2016; see review in Abdolahnejad & Liu, 2020). These models have been used in research to generate a wide range of images and can solve complex problems, such as predicting how changes will look in existing images (e.g., adding facial hair or reverting to an earlier age; Abdolahnejad & Liu, 2020). Nevertheless, generating a wide distribution of photorealistic faces of a unique ethnicity is still a rather complex task, as deep learning models often require large-scale datasets for training (Elasri et al., 2022), and current large-scale datasets are heavily skewed toward US-based content and images (e.g., Huang et al., 2008; Karras et al., 2019; Liu et al., 2015). Notably, with the quick advancement of new generative tools, their academic utility in the context of underrepresented populations might expand in the near future. Currently, there are no validated tools that accurately represent non-US populations, particularly those from the Middle East.

### Faces collected in the wild

Finally, researchers interested in generating a face dataset of specific underrepresented populations could collect multiple images of that population online through online browsers and social media (e.g., Bainbridge et al., 2013; Wang & Kosinski, 2018). This approach would create a large, diverse, and ecological database. However, such a database would nevertheless fail to serve basic research requiring images of multiple facial expressions of the same individuals, as well as research demanding any level of control over the production of the images (e.g., identical lighting, pose, and facial features across images). Additionally, this approach typically does not allow for the collection of complementary information about the individuals depicted, such as their demographic characteristics.

To conclude, approaches that involve generating artificial faces, as well as data collection in the wild, could be employed to create face datasets of underrepresented populations. However, as reviewed above, these different approaches have considerable limitations for research use, pointing to the importance of photographing real individuals under controlled procedures across various facial expressions.

## The present contribution

This paper presents a novel database that overcomes the abovementioned limitations: the Israeli Face Database (IFD). Specifically, the IFD represents a diverse non-US population, preserves ecological validity by presenting real face images, maintains high control over the image features, and provides multiple images per person. To access the database, researchers can follow the instructions in the OSF (https://osf.io/q2n7k/). Expression validation data and social rating data for each face identity are available in the OSF (https://osf.io/q2n7k/).

This database features the faces of real individuals from the Israeli population, which comprises a wide diversity of self-identified ethnicities (e.g., Jews, Druze, Bedouin, and Arabs) and ancestries^1^ (e.g., African, European, Middle Eastern, and Asian ancestries), of various religions (e.g., Jewish, Christian, and Muslim) and levels of religiosity. In recognition of the need for a more globally diverse science, efforts have begun toward creating face databases comprising non-White faces (Chen et al., 2021; Ma et al., 2015, 2021; Meyers et al., 2023; Pickron et al., 2024) and non-US participants (Courset et al., 2018; Lakshmi et al., 2021; Saribay et al., 2018). Despite this, ethnic groups outside the United States are still significantly underrepresented.

Israel’s population comprises a diverse range of ethnic groups and ancestries originating from many regions around the world. According to recent estimates (Smooha, 2023), approximately 82% of the Israeli population is Jewish, and 18% is Arab. Among the Jewish population, 43% trace their origins to various European countries (e.g., Russia, Romania, Poland, Germany), and 38% to countries in Asia and Africa (e.g., Morocco, Libya, Syria, Yemen, Iraq, Iran), with the remainder either of mixed heritage or originating from other regions. The Arab population includes 62.5% non-Bedouin Sunni Muslims, 22% Bedouins, 8% Christians, and 7.5% Druze, with the vast majority originating from the Middle East (e.g., Israel, Jordan, Syria, Saudi Arabia). Therefore, an Israeli population-based database could greatly help feature groups currently missing in existing face databases.

Moreover, although efforts have been made to correct the underrepresentation of ethnicities and ancestries, no database has documented various religions and levels of religiosity. As religion serves as a unique lens to the perception of the social world (Hunsberger & Jackson, 2005; Ysseldyk et al., 2010) and is the target of consequential stereotypes (Moon et al., 2021; Saroglou et al., 2011; Wright & Nichols, 2014), we see high value in expanding the variety of face identities to include this factor as well.

The IFD includes several facial emotional expressions of each photographed face, each validated by Israeli participants for alignment with the intended expression and authenticity. All faces of the IFD were photographed and processed using the same standardized procedure, ensuring high-quality stimuli suitable for academic research. In line with the growing need to provide more ecologically valid and authentic stimuli (Long et al., 2023), the IFD also includes naturally taken images of many of the face identities. The establishment of the IFD is an ongoing effort. Thus far, the database contains 78 facial identities. Additional facial identities are continuously being photographed and validated, with the ultimate goal for the IFD to include an even more comprehensive range of demographic diversity (e.g., Ethiopian ancestry and orthodox religiosity). This can be achieved as the database grows.

Moreover, and quite uniquely, the IFD contains face ratings from two different populations (i.e., Israelis and US residents) to assess the possible effects of cultural background and cultural affordances on social perceptions of human faces. *Cultural affordance* denotes the psychological responses, experiences, and meanings that tend to come to mind in a given culture and that depend on the various schemas, symbols, and lay theories that characterize that culture (Kitayama & Markus, 1999; Kitayama et al., 2006). In the context of face perception and judgment, people may respond differently to the same face due to cultural differences in the prevalence of specific social categorizations, personal goals, and the like. For example, trustworthiness judgments (Birkás et al., 2014; Schmid et al., 2022; Sofer et al., 2017) and masculinity and prosociality judgments (Jones et al., 2021; Shiramizu et al., 2024) can vary cross-culturally. To test the possibility that cultural affordance triggers cross-cultural differences in the perception and judgment of faces, we collected ratings from two cultures: Israeli and US-based participants. The ratings made by participants from these two cultures assessed various social judgments, such as the perceived ethnicity and typicality of the face and the extent to which different social attributes, such as dominance and trustworthiness, characterized the face. We selected US-based participants as the comparison culture, as this is the culture most frequently recruited to validate facial stimuli. Thus, it would be possible to compare the extent to which the ratings of Israelis differ from those of the typically recruited participants.

## Stimuli collection

### Method

#### Participants

Individuals photographed for this database were recruited from the Ben-Gurion University pool of subjects. Eighty-seven individuals agreed to be photographed for the database. Two participants were excluded following their request after the photographs were taken, and an additional seven were excluded due to nonremovable piercings or technical malfunctions that prevented the use of their photos. Of the remaining 78 participants, 25 identified as men, 51 identified as women, one as non-binary, and one as other. Participants’ ages ranged from 19 to 32 (*M* = 23.56, *SD* = 1.70). In addition, 66 participants identified as Jewish, six as Muslim, two as Christian, one as Hindu, and one as Pagan, while two did not identify with any religion.

As a measure of their ancestry, participants reported the countries of birth of each of their four grandparents. There were, in total, 38 countries of origin mentioned, with 72.73% of the participants reporting more than one country. The most frequently mentioned countries of origin (with at least one grandparent from those countries) were Israel (41.03%), Poland (25.64%), Morocco (16.67%), Germany (14.10%), Russia (14.10%), Romania (10.26%), Argentina (7.69%), Iraq (7.69%), Tunisia (7.69%), USA (7.69%), Ukraine (7.69%), and Yemen (7.69%).

#### Photo sessions

Upon arrival, participants received a consent form and an oral explanation, repeating its content. Participants were then asked to carefully read the consent form, sign it, and provide consent to have their photos taken and included in a database to be used in future experiments. The consent form also had explicit clauses asking for participants’ specific consent to (1) use their photos publicly (for example, on social media platforms), (2) present their photo alongside additional information (e.g., arbitrary political affiliation, trait, or evaluation) in future experiments, and (3) use another face image, provided by the participants, which was taken on another day. Participants could participate in the session even without providing consent to these three clauses; photos of such participants are marked in the materials accompanying the database. The forms were written in Hebrew and were translated on-site to English upon request. Afterward, participants wore a round-collar black T-shirt and were seated at a fixed distance of 170 cm from a Canon EOS 4000D camera with a Canon 50 mm f/1.8 STM lens. The camera was positioned on top of a SLIK 330DX tripod at a height of 122 cm. Next, participants removed any makeup, jewelry, and glasses and were asked to adjust their hair and the black shirt if necessary. Individuals with noticeable hair covers (e.g., a Hijab or a Jewish head cover) were asked to remove them.

All photo sessions took place in a room with no external windows. The room’s light was turned off during the photo sessions. Lighting was provided by a Godox LR180 ring light that was placed on a Porta Brace PB2600 tripod in front of the camera, and a Godox LED500C Video light that was attached to a PB2600 tripod and placed behind and to the side of the participant, projecting light on a green roll-up screen that was used as the background in the session. Participants were instructed to make facial expressions that included neutral (with and without a mask), happy (with open- and closed-mouth smiles), and angry expressions while maintaining an upright and straight head position. In later sessions (after the 50th participant), participants also posed a sad expression. Each facial expression was photographed in up to three phases. In the first phase, participants were asked to think of something or someone that made them feel the emotion being photographed at the time. In the second phase, participants were shown illustrations of the relevant facial expression taken from Langner et al. (2010) and were asked to imitate that expression. In the third phase, participants were instructed to think about what made them feel the emotion and try to imitate the expression as they did in the second phase. In later sessions, the imitation step in the second phase was omitted to save time. At the end of the photo session, participants were asked to fill out a demographic questionnaire. In the questionnaire, participants were asked to provide their gender identification, age, marital status, number of children, education, employment status, grandparents’ country of birth, personal identity (e.g., Israeli or Palestinian, Orthodox Jew), religion, level of religiosity, and socioeconomic status. Finally, we asked participants to rate their difficulty expressing each expression on a scale of 0–100 (0 = *not at all difficult*, 100 = *very difficult*). Each session lasted approximately 1 h. The results of the demographic questionnaire and the difficulty ratings are available with the face database.

##### Stimuli standardization

**Fig. 1:**
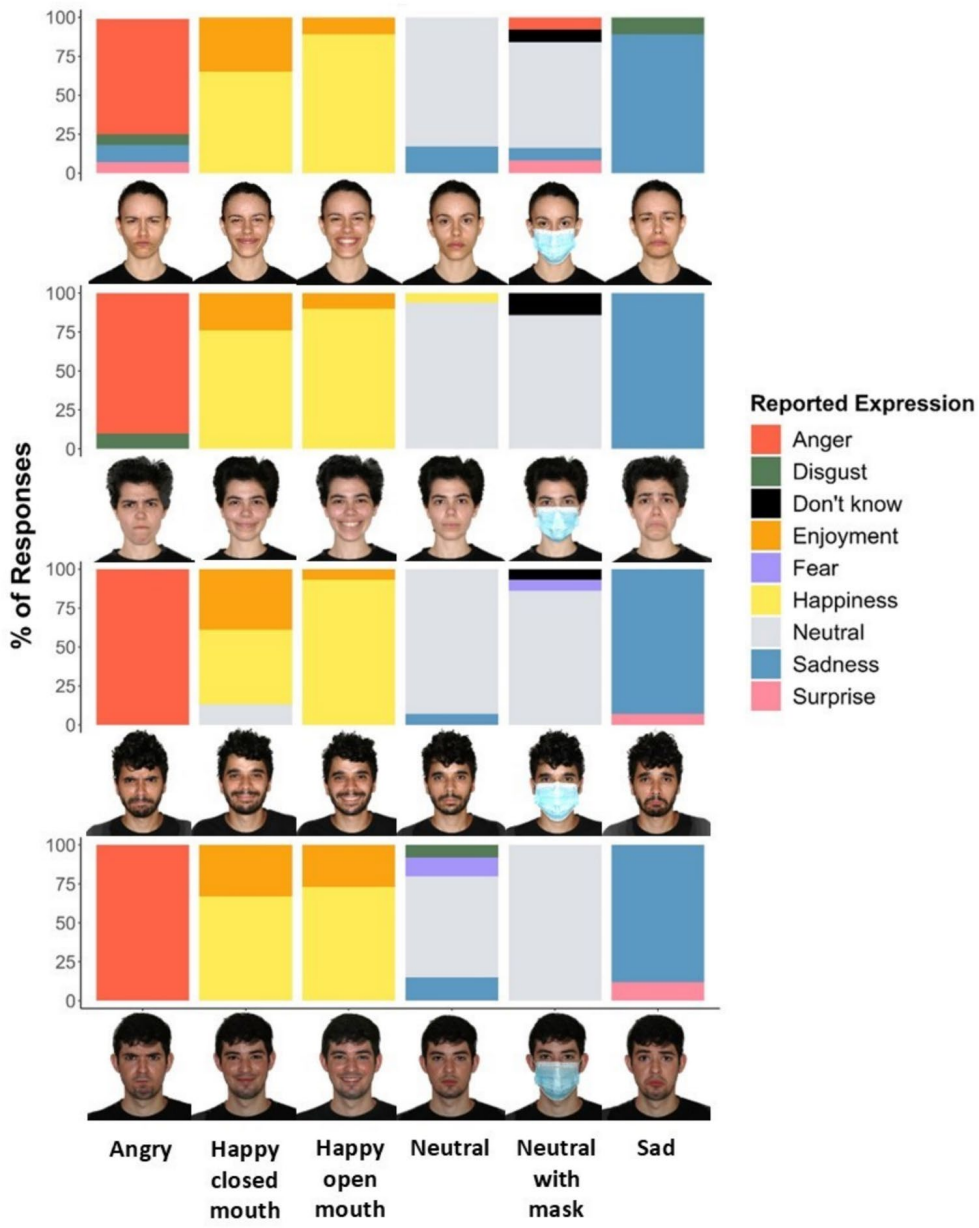
Examples of IFD face images and distribution of expression recognition responses to six expressions. See an accessible version in the SOM

 One image of each facial expression was selected based on the quality and believability of the expression and the quality of the image (including the positioning of the head and the tightness of the shirt around the shoulders of the target). Images were selected by an inter-rater procedure that included 2–4 experimenters who reached complete agreement for each image. The images were then edited using GIMP [version 2.10.22] (The GIMP Development Team, 2019). The green background was converted to white, and the images were straightened and resized so that the core facial features were relatively equal in size across face identities. To ensure standardization, core features were aligned using a standard rectangle in which the top vertices met the inner edge of the eye, and the bottom edge met the top of the upper lip. All images were cropped and resized so that their final size was 4,075 × 3,455 pixels. Following the execution of Study 1, all stimuli were also realigned. Specifically, images were realigned using the *auto_delin()* and *align()* functions of the webmorphR R package (DeBruine, 2022) so that, within all facial expression images of each face identity, all images were centered to the same spatial location with the face’s eyes as a reference point. Images were cropped again to remove any redundant patch filling around the image caused by the alignment procedure. Figure 1 shows sample stimuli following this procedure.

## Overview of the studies

To characterize the images in the IFD, we designed two studies. The purpose of Study 1 was to validate the facial stimuli for the facial expression enacted and its authenticity. Following the establishment and validation of the database, Study 2 was designed to obtain norms (ratings) regarding several social judgments from the faces (perceived ethnicity, typicality to that ethnicity, perceived social attributes) and to investigate whether these ratings varied between cultures. All data and analysis codes and the Supplementary Online Materials (SOM) are available online at https://osf.io/q2n7k/.

## Study 1: Validation of the IFD

The purpose of Study 1 was to validate the basic features of the faces included in the IFD, such as the facial expression we intended to capture, and to obtain norms for each facial expression. Validation followed the indices reported in a previous commonly used face database (Radboud Faces Database; Langner et al., 2010). As detailed below, there were two facial expression validation (i.e., norming) rounds, each targeting a different subset of faces from the database.

### Methods

#### Participants

We aimed to achieve at least 30 valid ratings for each face identity. To ensure this sample size while accounting for potential dropouts, we planned to recruit at least 700 participants (350 in each round). In the first validation round, 437 Israeli participants were recruited to an online study in which each image was rated across different dimensions. All participants were recruited via Panel HaMidgam (https://www.midgampanel.com/), an online platform for recruiting Israeli participants. Twenty participants from the original sample did not complete the study, and 97 participants were excluded for failing one or more of the four attention checks we included in that study. Two additional participants were excluded because the average ratings they provided exceeded the interquartile range more than four times across measures. Thus, 318 participants were included in the analysis (164 women, 153 men, and one participant who selected “other” as their gender identity; *M*_age_ = 30.24, *SD*_age_ = 5.48; percent Jewish Israelis = 97.80%). In the second validation round, 609 participants were recruited (via Panel HaMidgam). Of those participants, 53 did not complete the task to its end, and 248 were excluded for failing one or more of the five attention checks we included in that study. Ten other participants were excluded because the average ratings they provided exceeded the interquartile range more than four times across measures. Thus, 298 participants were included in the analysis (149 women, 149 men; *M*_age_ = 42.54, *SD*_age_ = 16.00; percent Jewish Israelis = 84.23%).

#### Procedure

After a brief textual instruction section, participants in each of the two rounds were presented with one image at a time and were asked to select which one of eight expressions (happiness, enjoyment, surprise, anger, disgust, sadness, fear, and neutral feeling) most closely corresponded to the expression displayed in the image. The eight possible expressions were fixed across images. Then, participants rated the intensity, clarity, and honesty of the expression appearing in the image on a visual analog scale (VAS), ranging from 0 (*not at all*) to 100 (*very much*). Finally, participants rated the valence of the expression on a VAS ranging from 0 (*very negative*) to 100 (*very positive*).

To keep the experimental session relatively brief, each participant saw the images of four face identities from our database. Participants in the first round rated a pool of 40 face identities, which were pictured posing five different expressions. Participants in the second round rated another pool of 38 face identities that were pictured posing six different expressions.

Participants in the first round also saw and rated the images of two face identities from the Chicago Face Database (CFD, Ma et al., 2015) with neutral, happy (closed- and open-mouthed), and angry expressions. These ratings served to assess the convergent validity of the face images. Each image from our database was rated, on average, 31.80 times (*SD* = 1.98) or 36.70 times (*SD* = 3.15) on the first and second samples, respectively.

##### Attention checks

We included four attention check items in the first validation round and five items in the second round. The first item in both rounds appeared immediately after the instructions, where participants were instructed to select a specific response in a forced-choice question. The remaining items were designed to resemble the selection of expressions, featuring face identities that were not included in the analysis or the database. After participants responded, they were shown a face image—either the same face or a different one—and asked to indicate whether it was the last face they had seen. To be included in the final sample, participants were required to pass all attention checks.

#### Analysis strategy

First, we wanted to ensure a match between the images’ intended expression and the expression chosen by the participants. For this purpose, we conducted a chi-square test, examined how the responses of expression recognition were distributed for each facial expression, calculated the percentage of correct expression recognition, and the proportion of faces whose expression was recognized correctly by at least two-thirds of the participants. Second, to estimate the quality of the enacted expressions, we calculated the mean intensity, clarity, honesty, and valence ratings per target and participant. This also allowed us to identify extreme ratings of a specific target or participant. Third, we conducted a Bayesian one-sample *t*-test to ensure the neutral expressions’ valence was not significantly different from 50 (i.e., neutral valence). Finally, to estimate the expressions’ validation relative to a commonly used face database, we conducted a set of paired *t*-tests to compare all the ratings of IFD images to those of CFD images, as rated by the same participants from the first validation. All comparisons were corrected for false discovery rate (FDR; Benjamini & Hochberg, 1995).

### Results

Average expression ratings of each face identity in the database (across both validation rounds) are presented in the accompanying file “IFD_expression_ratings_per_face_Exp1.csv,” also available at https://osf.io/q2n7k/.

#### Matching of intended and chosen expression

The chi-squared test yielded a significant result in the first expression validation round for both IFD faces, χ^*2*^(28) = 9,689.90, *p* < 0.001, and CFD faces, χ^*2*^(21) = 3,758.10, *p* < 0.001. The second expression validation round (which additionally included sad expressions) yielded similar results, χ^*2*^(60) = 14,748, *p* < 0.001. Namely, in both rounds, expression responses were distributed similarly to the actual expressions, suggesting they were recognized correctly. Table 1 presents the proportion of face identities whose expressions were correctly recognized by at least 66.7% of participants and the mean accuracy rates for recognition of each expression across face identities.

**Table 1:** The proportions of correct expression recognition, mean ratings, and standard deviations for each facial expression in the IFD in Study 1

Expression	Valence		Intensity		Clarity		Honesty		Proportion of correct recognition		≥ 0.667 recognized correctly	Number of raters
	*M*	*SD*	*M*	*SD*	*M*	*SD*	*M*	*SD*	*M*	*SD*		
Anger	19.37	19.15	65.95	25.84	64.62	28.25	55.41	30.32	0.60	0.49	0.52	616
Happiness, closed mouth	75.84	21.02	63.41	24.59	66.08	25.95	60.76	28.79	0.77	0.42	0.94	616
Happiness, open mouth	86.39	17.62	79.29	21.21	80.64	21.89	74.80	25.59	0.85	0.35	0.97	616
Neutral without mask	44.61	19.06	63.78	25.97	61.24	27.84	66.08	25.32	0.63	0.48	0.70	616
Neutral with mask	46.33	18.72	55.02	26.64	40.12	28.83	51.06	28.05	0.61	0.49	0.61	616
Sadness	22.40	20.07	69.98	25.56	72.77	25.56	56.61	31.59	0.79	0.41	0.79	298

Scores represent the means and standard deviations of the mean scores of each face.

We considered both “happiness” and “enjoyment” as correct recognitions of happiness (with either open or closed mouth).

≥ 0.667 recognized correctly = the proportion of face identities whose expressions were correctly recognized by at least 66.7% of participants.

#### Expression ratings

Figure 1 presents examples of the distribution of expression recognition responses to all six expressions of four face identities from the IFD. Table 1 shows the mean ratings and standard deviations for each facial expression in the IFD (anger, sadness, happiness with an open mouth, happiness with a closed mouth, neutral expression, and neutral with a mask). Importantly, valence ratings of the non-neutral expressions correspond to the expected ratings. Specifically, valence was lower than 50 for anger, *t*(2351) =  − 77.56, *p* < 0.001*,* and sadness, *t*(1144) =  − 46.54, *p* < 0.001, and higher than 50 for happiness with open mouth, *t*(2408) = 101.38, *p* < 0.001, and closed mouth, *t*(2389) = 60.09, *p* < 0.001. Table S1 in the SOM shows the rating data for CFD faces, as rated by participants of the first round.

##### Valence of neutral images

 Contrary to our expectations, there was extremely strong evidence that ratings of neutral facial expressions were significantly lower than 50 for both images without a mask (*M* = 44.50, *SD* = 19.03), BF_10_ > 1,000, and images with a mask (*M* = 46.60, *SD* = 18.48), BF_10_ > 1,000. Although there were not enough CFD face identities to examine significance, a similar pattern emerged for the ten CFD neutral images (*M* = 40.41, *SD* = 6.06). Thus, it appears that neutral images were judged as somewhat negative across databases.

##### Comparison between IFD and CFD ratings

 Ratings in the first validation were not significantly different for IFD and CFD faces, except for the valence of images presenting a happy expression with a closed mouth, which was higher among IFD (*M* = 73.65, *SD* = 14.70) than among CFD (*M* = 71.26, *SD* = 17.92) faces, *t*(317) = 3.17, *p* = 0.026, Cohen’s *d* = 0.15, honesty of the neutral faces, which was higher among IFD (*M* = 65.79, *SD* = 18.67) than CFD (*M* = 63.13, *SD* = 20.69) faces, *t*(317) = 2.72, *p* = 0.023, Cohen’s *d* = 0.15, intensity of angry faces, which was lower among IFD (*M* = 62.31, *SD* = 18.18) than CFD (*M* = 65.08, *SD* = 20.29) faces, *t*(317) =  − 2.81, *p* = 0.023, Cohen’s *d* =  − 0.16, and intensity of happy faces with closed mouth, which was higher among IFD (*M* = 59.26, *SD* = 18.27) than CFD (*M* = 56.65, *SD* = 20.90) faces, *t*(317) = 2.78, *p* = 0.023, Cohen’s *d* = 0.16. A complete report of the results of this set of comparisons is presented in the SOM.

### Discussion

Study 1 aimed to validate the expressions posed in the images of the IFD by testing whether displayed expressions accurately represented their intended expressions. The results supported this objective with both happiness expressions, sadness, and neutral-without-mask expressions, with participants demonstrating relatively high accuracy in expression recognition across the different emotion categories. The anger and neutral-with-mask expressions were recognized less accurately, aligning with the expression recognition rates of CFD faces, which were also substantially lower than the happiness expressions. The overall correct recognition was further corroborated by chi-squared analyses, confirming that the expression responses were distributed similarly to the intended facial expressions in the IFD.

The expressions’ valence ratings provided additional validation, with scores being relatively high for positive expressions (happiness) and low for negative expressions (anger, sadness). However, contrary to our expectations, valence ratings of the neutral expressions were somewhat negative. This finding aligns with previous research indicating that neutral faces are often perceived as slightly negative (E. Lee et al., 2008). One potential explanation for this phenomenon is that neutral expressions are relatively uncommon in typical social interactions, making them appear somewhat unnatural or potentially negative (Phillips et al., 1997, 2004).

Importantly, we found substantial similarity between ratings of corresponding facial expressions in the IFD and the CFD. This cross-database consistency provides strong evidence for the IFD’s validity. Together, these findings indicate that the IFD is well suited to various research paradigms involving facial stimuli, particularly studies investigating emotional expression processing.

## Study 2: Social attribute norms and a test of cultural affordance effects

The purpose of Study 2 was to collect norming data regarding social judgments made based on the faces of the IFD and to compare the ratings obtained from two different cultures (Israel and the United States). We collected ratings of several social attributes frequently used in impression formation research (e.g., Hester et al., 2021; Ma et al., 2015, 2021). We additionally assessed perceived masculinity and femininity (adapted from Chen et al., 2021; Saribay et al., 2018), perceived ethnicity of the face and how typical the face looked for each chosen ethnicity, perceived nationality, street typicality (i.e., how often the individual may appear within the participants’ social environment), and perceived age.

### Method

#### Participants

We aimed to achieve at least 40 valid ratings for every social trait of each face identity from each sample. To ensure this sample size while accounting for potential dropouts, we planned to recruit at least 1440 participants from each sample. Participants were recruited to an online study focused on face perceptions and were asked to make several judgments regarding the faces they were shown. Israeli participants were recruited from the Panel HaMidgam platform, and US participants were recruited from Prolific (Palan & Schitter, 2018; https://www.prolific.com/).

##### Israeli sample

 Of the 1,921 participants who started the study, 1,444 completed all study parts. Of these, 108 participants failed all attention checks. The final sample included 629 men, 698 women, and nine non-binary/other (mean age = 43.07; *SD* = 15.26, percent Jewish Israelis = 99.03%). Our data also contain participants’ responses regarding their religiosity level and education (see the SOM for a full description).

##### US sample

Of the 1534 participants who started the study, 1445 completed all study parts. Of these, 246 participants failed all attention checks. The final sample included 582 men, 590 women, and 27 non-binary/other (mean age = 40.76; *SD* = 13.80; 61.05% White, 21.10% Black, 3.84% Eastern Asian, 3.00% Hispanic/Latinx and White, 2.59% Hispanic/Latinx, 1.08% White and Black, 6.89% other). Our data also contain participants’ responses regarding their religious identity and education (see the SOM for a full description).

#### Procedure and materials

To reduce the study length, participants were randomly assigned to rate 26 out of 78 faces. Face sets were created by dividing the 78 faces into three subsets of 26 faces in two different orders (six subsets in total); each participant saw only one of the six possible subsets. Furthermore, we created two randomization conditions, Condition A and Condition B, each with a separate subset of six of the 12 social traits rated in this study. Each participant in each condition rated all 26 faces on one (randomly selected) of the relevant six traits, followed by two questions assessing perceived masculinity and femininity, a question assessing the perceived ethnicity/ethnicities of the person whose face was presented, and a question about the perceived typicality of the face for each of the indicated ethnicities. In Condition A, participants were additionally asked either one (US sample) or two (Israeli sample) questions about the perceived nationality of the person. In Condition B, the perceived nationality questions were replaced with a question about the overall typicality of the person and a question about the person’s perceived age. In both conditions, participants were randomly assigned either to start by rating the perceived social trait of all faces followed by rating the additional dimensions, or to finish with the trait rating. Participants completed three attention check items: one following the trait rating, one following the perceived nationality (Condition A) or perceived age (Condition B) questions, and one in the middle of the demographic questionnaire.

##### Perceived social traits

The social traits rated were *aggressiveness*, *attractiveness*, *care*, *self-confidence*, *dominance*, *emotional stability*, *intelligence*, *meanness*, *responsibility*, *friendliness*, *trustworthiness*, and *weirdness*. We chose these traits because they are frequently used in impression formation research (e.g., Hester et al., 2021; Ma et al., 2015, 2021). For each face image, participants were asked to rate, based on observable facial features, the degree to which a specific trait characterized the person appearing in the image. Participants were asked to rate the person compared to other people of the same gender and ethnicity as the featured person. Participants made their ratings on a visual analog scale (VAS) ranging from − 100 (*not at all characterized*) through 0 (*to an equal extent*) to 100 (*very characterized*).

##### **Perceived masculinity and femininity**

For each face image, participants were asked to rate the extent to which they thought the person appearing in the image was characterized by masculinity and femininity. Participants made both ratings on a VAS ranging from − 100 (*not at all characterized*) through 0 (*to an equal extent*) to 100 (*very characterized*).

##### Perceived ethnicity

For each face image, participants were asked to select the ethnicity/ethnicities that were most characteristic of the person appearing in the image. The options were *Northern/Western/Central Europe*, *Southern Europe*, *Eastern Europe*, *Middle East*, *West Africa*, *North Africa*, *Central/Eastern/Southern Africa*, *Central and South America*, *North America*, *South Asia*, *East and Southeast Asia*, *West Asia*, *Pacific Islands and Hawaii*, *Other (please specify)*. Representative countries were provided as relevant examples of these options to improve clarity. Participants could select more than one response option.

##### Perceived typicality of ethnicity

For each face image, participants were asked to rate the extent to which the face seemed typical of the ethnicity/each of the ethnicities they selected on a VAS ranging from − 100 (*not at all typical*) through 0 (*to an equal extent*) to 100 (*very typical*).

##### Perceived nationality

This measure differed for the Israeli and US samples to examine whether both samples could consider the faces as members of their national group. For the Israeli sample, the measure included two items. One item assessed perceived Israeli-Jewish nationality,^2^ as follows: *To what extent does this person look like a person of Jewish nationality living in Israel, as compared to a person of some other nationality?* Response options were *another nationality for sure*, *probably another nationality*, *not sure*, *probably a person of Jewish nationality*, *a person of Jewish nationality for sure*. A second item assessed Israeli-Arab nationality as a rival, yet visually similar, national group that was in frequent contact with the participants’ group (Bar-Tal & Teichman, 2009): *To what extent does this person look like a person of Arab nationality living in Israel, as compared to a person of some other nationality?* Response options were *another nationality for sure*, *probably another nationality*, *not sure*, *probably a person of Arab nationality*, *a person of Arab nationality for sure*. For the US sample, this measure included a single item, assessing perceived American nationality: *To what extent does this person appear to be a person residing in the U.S. with an American nationality, compared to a person from another nationality?* Response options were *another nationality for sure*, *probably another nationality*, *not sure*, *probably residing in the U.S. with an American nationality*, *residing in the U.S. with an American nationality for sure*.

##### Street typicality of the face

 For each face image, participants were asked to rate how likely it would be to meet a person similar to the person appearing in the image on the street. Participants made their ratings on a VAS ranging from − 100 (*not at all likely*) through 0 (*to an equal extent*) to 100 (*very likely*).

##### Perceived age

 For each face image, participants were asked to estimate the age of the person appearing in the image. Participants made their judgments on a VAS ranging from 18 to 100.

##### Attention checks

The studies included three attention check items. The first item was designed to appear similar to the items assessing perceived social traits. Participants were required to respond with a specific number on the VAS. The second item was designed to resemble the perceived nationality item (Condition A) or the perceived age item (Condition B) while requiring the participants to respond with a specific number on the VAS. The final item was designed to resemble a demographic question. Participants were required to select the response option displaying the sum of specific numbers. Participants had to pass all three attention checks to be included in the final sample.

#### Analysis strategy

The main aim of the analyses was to explore the differences and similarities between the ratings given by US and Israeli participants. First, we conducted a set of one-sample *t*-tests to examine which social ratings were different from 0. Notably, we examined the ratings of US and Israeli participants separately; we further separated the analysis of the masculinity and femininity ratings for male and female faces. All comparisons were corrected using FDR (Benjamini & Hochberg, 1995). Second, we examined whether the social ratings differed between US and Israeli participants by conducting a set of two-sample *t*-tests, corrected using FDR. Third, we examined whether the ratings of US and Israeli participants were correlated at the face level by conducting a set of Spearman correlations. Fourth, we conducted a set of Levene’s tests to examine whether the ratings given by Israeli participants were more heterogeneous than those provided by US participants. Fifth, we examined how the perceived nationality responses were distributed among US and Israeli participants. Finally, we examined how the perceived ethnicity responses were distributed among US and Israeli participants.

### Results

**Fig. 2:**
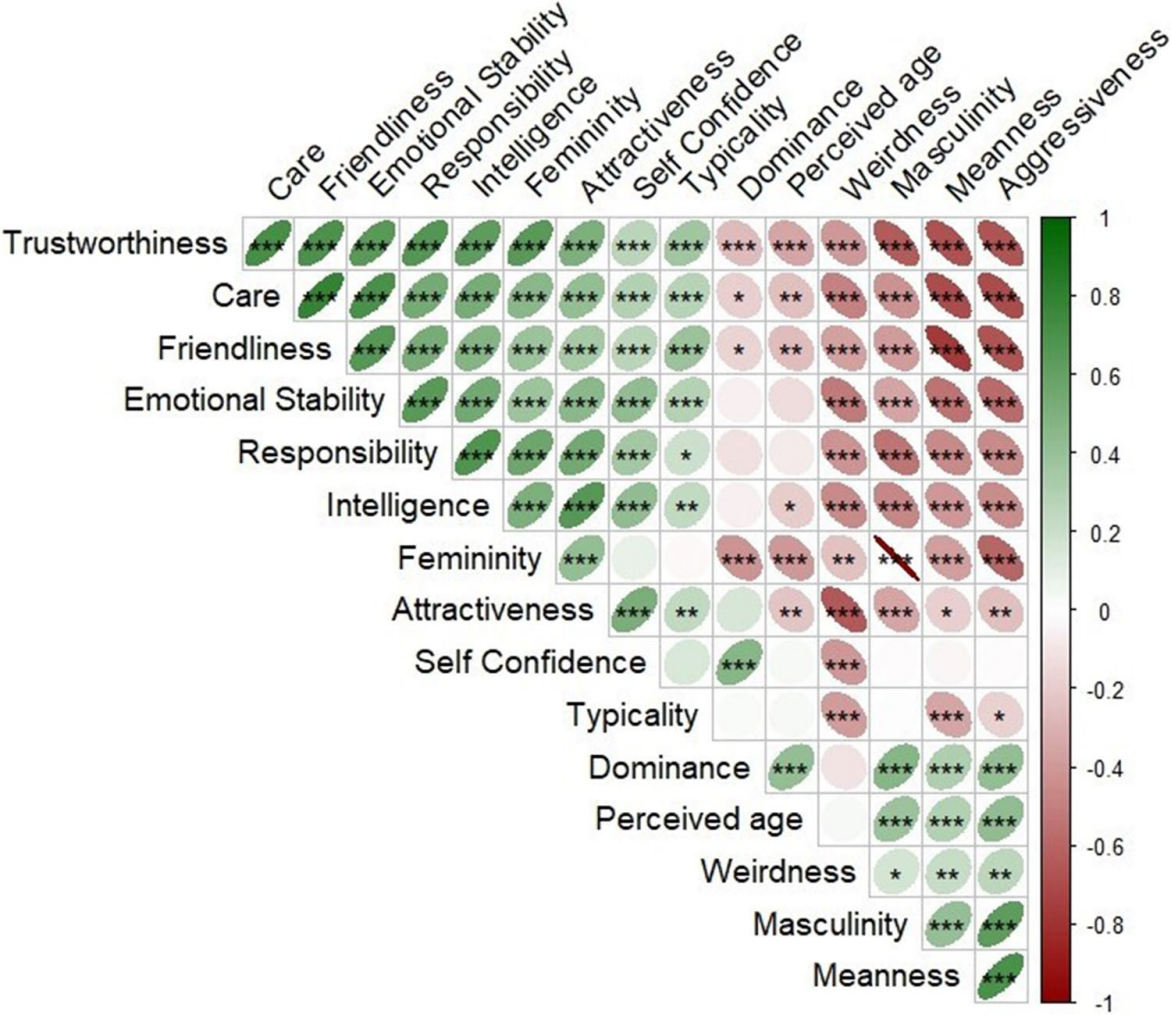
Cross-correlation matrix of average face ratings across multiple traits across both the Israeli and the US-based samples. The matrix shows the Spearman correlation coefficients between each pair of traits. All correlations were computed using the mean ratings for each face across all participants. * *p* < 0.05, ** p < 0.01, *** *p* < 0.001. Matrices for each sample separately are presented in the SOM

The average ratings for each face identity (across each sample separately and across both samples) are presented in the accompanying file “IFD_social_ratings_Exp2.xlsx,” available at https://osf.io/q2n7k. Figure 2 presents a cross-correlation matrix of average face ratings across multiple traits across both samples.

#### Comparing trait ratings between the US and Israeli samples

All ratings were significantly different from 0 (all corrected *p*s < 0.029), except for the friendliness ratings given by US participants, *t*(77) = 1.42, *p* = 0.166, and attractiveness ratings given by Israeli participants, *t*(77) =  − 1.35, *p* = 0.181. A complete report of these tests is presented in the SOM.

**Fig. 3:**
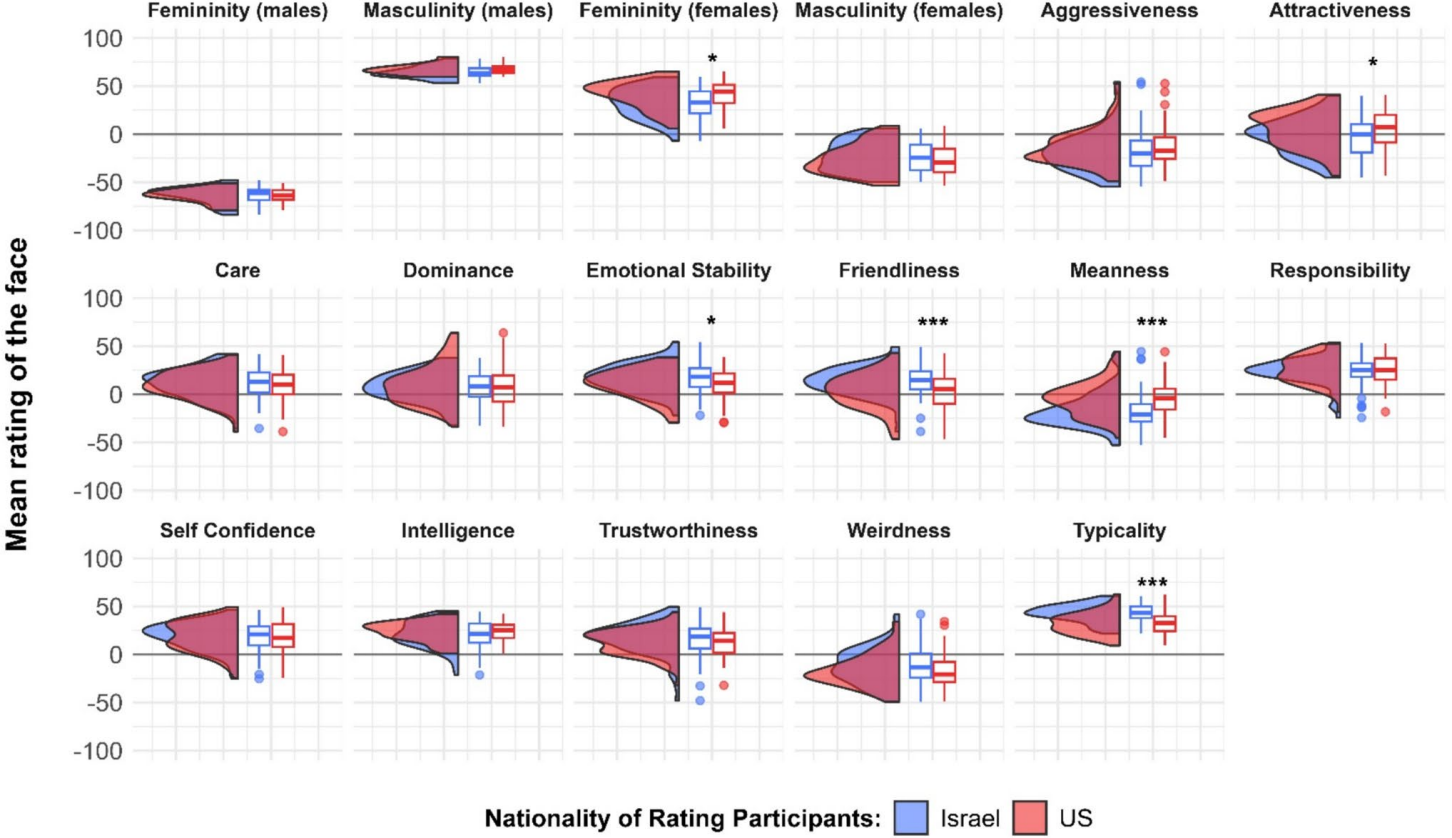
Distributions of social ratings, as rated by US and Israeli participants in Study 2. * *p* < 0.05; *** *p* < 0.001. See an accessible version in the SOM

Figure 3 presents the distributions for rated traits in the US and Israeli samples in Study 2, as well as the results of the comparisons between the two samples. Ratings did not differ between the samples, except for the following: US participants rated the same faces as more attractive, *t*(153.95) =  − 2.82, *p* = 0.018, and more mean, *t*(153.87) =  − 4.55, *p* < 0.001, than Israeli participants did, and rated the female faces as more feminine, *t*(102.72) =  − 3.00, *p* = 0.015; Israeli participants rated the faces as more typical, *t*(149.73) = 6.47, *p* < 0.001, emotionally stable, *t*(153.57) = 2.79, *p* = 0.018, and friendly, *t*(147.62) = 4.10, *p* < 0.001, than US participants did. A complete report of all the rating comparisons is presented in the SOM.

#### Trait rating correlations between US and Israeli samples

Across all rated traits, there were significant correlations between the mean ratings of each face as rated by US and Israeli participants (all *r*s > 0.49, all *p*s < 0.001), indicating consensus in impressions across cultures. A complete report of the correlation results is presented in the SOM.

#### Differences in rating heterogeneity between US and Israeli samples

Another source of difference between the rating samples could have been the heterogeneity of their responses to the same faces, which might have occurred due to the greater familiarity of the faces to Israelis than to US-based participants. A set of Levene’s tests showed that intelligence ratings were more heterogeneous among Israeli than US participants, *F*(1,154) = 7.87, *p* = 0.005, and dominance ratings were more heterogeneous among US than Israeli participants, *F*(1,154) = 4.83, *p* = 0.029. No significant differences in heterogeneity were found in any other ratings, indicating that although the faces belonged to the same population as the Israeli rating participants, this did not cause their responses to the faces to be more similar than those of US-based participants. A complete report of the tests’ results is presented in the SOM.

#### Perceived nationality and perceived ethnicity

**Fig. 4:**
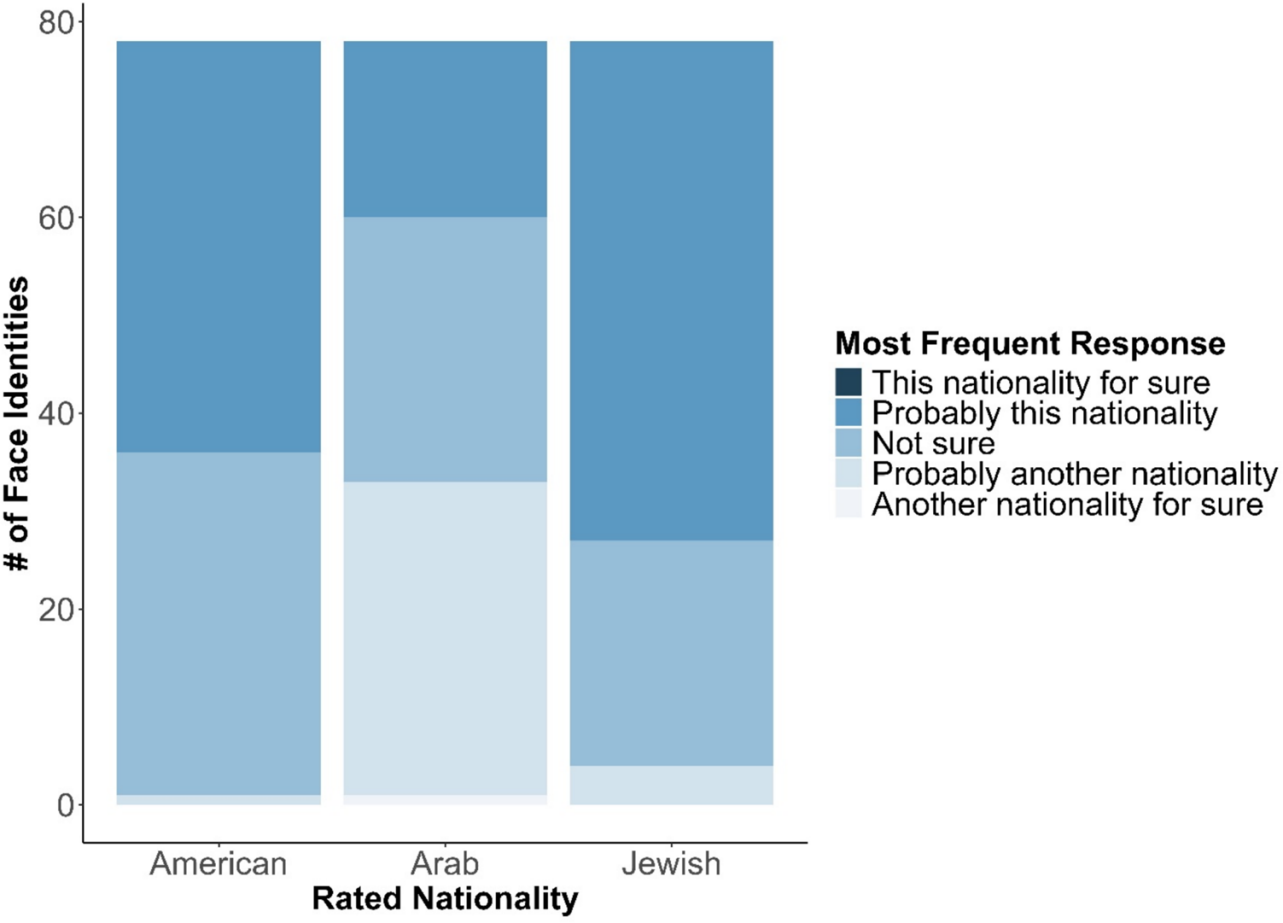
Distribution of the most frequent nationality ratings of faces. US participants rated the perceived American nationality, and Israeli participants rated both the perceived Arab–Israeli and Jewish-Israeli nationalities

**Fig. 5:**
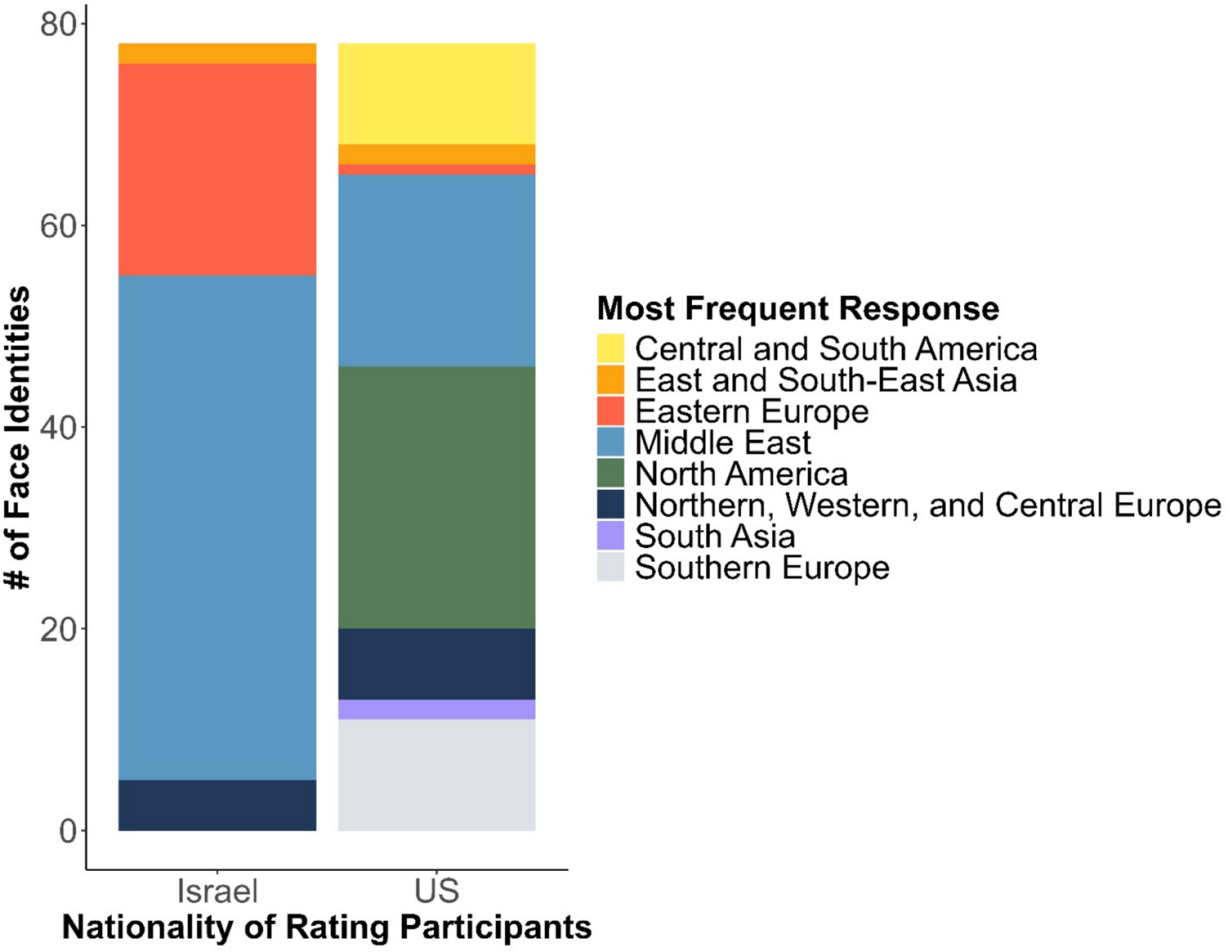
Distribution of the most frequent ethnicity categorizations of faces, as categorized by US and Israeli participants. See an accessible version in the SOM

Figures 4 and 5 present the distributions of the most common responses of perceived nationality and perceived ethnicity, respectively. Both US and Israeli participants rated the majority of the faces as probably belonging to their nationality: 42 faces were most frequently rated by US participants as *probably American*, while 51 faces were most frequently rated by Israelis as *probably Jewish from Israel*. Interestingly, 35 faces received both responses (i.e., they were rated both as *probably American* by US participants and as *probably Jewish from Israel* by Israeli participants). Israeli participants rated only 18 faces as *probably Arabs from Israel*; five of these 19 faces were also rated by the same sample as *probably Jewish from Israel*.

Ethnicity ratings were more diverse among the US than the Israeli participants. Analyzing the most frequent ethnicity categorizations for each face showed that only four such categories were given by Israeli participants (with 50 faces most frequently categorized as *Middle Eastern* and 21 faces as *Eastern Europeans*), compared to eight categories given by US-based participants (with only 19 faces most frequently categorized as *Middle Eastern* and one face as *Eastern European*). For both samples, all chosen categories were also rated higher than 0 on the ethnicity-typicality scale, confirming that all ethnicities were chosen because the participants believed them to accurately represent the faces’ ethnicities.

## General discussion

In recent years, it has become clear that overreliance on White and Western populations limits the scope of findings in psychological research and that increasing the diversity of studied populations and stimuli to match diverse living experiences is critical. Given the prominence of faces in social experiences, diversifying stimuli also includes expanding the variety of face stimuli used in studies. The central aim of the IFD is to address this need by providing a high-quality face database that represents a range of ethnicities, ancestries, and religions. To make it suitable for various research questions and methods, the IFD features multiple facial expressions per identity alongside social norming information available for researchers using the database.

We conducted two studies to assess the IFD’s suitability for psychological research. In Study 1, we validated the authenticity and face validity of enacted facial expressions within the same cultural group as the faces depicted. In Study 2, we compared social judgments of the face identities given by participants from the same culture (Israel) to those from a different, extensively studied culture (the USA).

For most social traits assessed, we found consistency in face judgments across the two cultures, evident both at the group level, with similar mean ratings, and at the individual level, with strong correlations between each face’s ratings across the cultures. This similarity aligns with previous findings showing that in the absence of group labels or extreme visual cues that imply group categorization, social ratings are influenced mainly by individual differences of the participants and the rated faces (Albohn et al., 2024; Hehman et al., 2017; Saribay et al., 2024).

However, some notable exceptions emerged from this overall similarity. Although typicality ratings were above the midpoint in both cultures, Israeli participants rated faces as significantly more typical than US participants. This aligns with the composition of the database, which features individuals residing in Israel. The difference in perceived typicality may help explain other observed cross-cultural variations. Typicality is strongly associated with perceived familiarity (Bartlett et al., 1984; Halberstadt & Rhodes, 2003), which is known to foster positive feelings toward faces, enhance liking, and make them seem safer compared to unfamiliar faces (Zebrowitz et al., 2007). Through its connection with perceived familiarity (Sofer et al., 2015), Israeli participants may have judged the faces as friendlier and more emotionally stable while perceiving them as less mean compared to US participants. Supporting this interpretation, we found positive correlations between mean ratings of typicality, emotional stability, and friendliness, alongside negative correlations between these ratings and perceived meanness. This pattern was consistent across both US and Israeli samples.

Another notable difference was the higher attractiveness ratings given by US participants, possibly related to their tendency to rate the female faces in the database as more feminine (Pereira et al., 2019). This may also be linked to typicality, as more attractive individuals are often perceived as less typical (Alley & Cunningham, 1991; DeBruine et al., 2007).

Interestingly, both US and Israeli participants tended to categorize most face stimuli as members of their own nationality (either US residents or Jewish Israelis). This finding may explain the similar trustworthiness ratings across cultures, as participants primarily perceived faces as members of their own society (Hong & Freeman, 2024). Nevertheless, the perceived ethnicity distributions differed markedly between samples, suggesting that the social rating similarities were not driven by universally shared perceptions of ethnic characteristics.

Together, these differences illustrate two manifestations of social affordance in person perception. First, they show that judgments of social traits can be shaped by how well a face aligns with a group’s schema of a typical member. When a face more closely matches this schema, the person is more likely to be attributed traits believed to characterize the group (e.g., high friendliness, low meanness). Second, they reveal that different cultures may interpret the same facial phenotypes in distinct ways, potentially based on culturally available representations of ethnic appearance (e.g., what does a Latinx face look like? What does a Middle Eastern face look like?). Relative to these representations, a face may be categorized as belonging to one ethnic group in one culture and to a different group in another.

Notably, the IFD offers multiple opportunities to explore cultural affordances that were not addressed in the current study. For instance, the present work did not examine whether individuals from different cultures vary in how they perceive and interpret the facial expressions included in the database. Given previous research suggesting that facial expressions may be perceived differently across cultures (Gendron et al., 2014; Jack et al., 2012), this could be a promising direction for future investigation.

Our findings demonstrate that the IFD is suitable for expanding diversity in face perception research, as faces in this database are associated with a broader range of ethnicities and perceived as less typical among US participants while still judged relatively similarly across different cultural backgrounds. This pattern of cross-cultural similarities and differences underscores the complex interplay between universal and culturally specific aspects of face perception, emphasizing the importance of cultural context in this research.

We propose that the IFD is well suited for various fields of social-psychological research. It can be particularly beneficial for studies on facial expression processing across ethnicities, social perception of Middle Eastern individuals, impression formation of faces varying in ethnic and social characteristics (e.g., dominance, trustworthiness, masculinity, femininity), and generalization across multiple appearances of the same identity, such as across different facial expressions or from a controlled to a naturalistic image.

The current version of the IFD does have some limitations. First, it has yet to be tested with participants outside Israeli and US populations, so it is unclear how other, less-studied populations would judge and perceive the faces. Second, the database contains posed rather than spontaneous expressions performed by nonprofessionals. Research has shown that posed expressions are often perceived and recognized differently from natural ones (Aviezer et al., 2012; Sauter & Fischer, 2017). This may influence participants’ responses, potentially making them less accurate or leading to judgments of the expressions as less intense and more sincere than ecological stimuli. Third, we have so far collected data on the images of 78 faces. As of this stage, 168 more face identities are included in the database that have not gone through norming yet, but their face images are available. We are actively expanding the database, aiming for greater diversity, including individuals of more ethnicities within Israeli society and over a wider age range. We also aim to better represent Israel’s religious minorities and varying religiosity levels.

In summary, as part of the ongoing effort to diversify psychological science, we offer a new, high-quality face database of real individuals from ethnic groups that have been underrepresented in social research stimuli. We believe that such diversification of face stimuli will enable better generalizability of studies’ findings in two ways: first, by examining participants’ performance in response to more facial phenotypes, and second, by examining the performance of non-US participants reacting to faces of their own ethnicities. Incorporating the IFD in future studies represents a step toward more generalizable—and thus better—science.

## Supplementary Information

Below is the link to the electronic supplementary material.

Supplementary file1 (DOCX 1031 KB)

## Data Availability

The data and materials for both studies are available on the Open Science Framework (https://osf.io/q2n7k/).

## Footnotes

1 Ancestry was coded according to the participant’s response to a question about the birthplace of each of their grandparents.

2 Although Judaism is a religion, it is often also considered a nationality (e.g., Yakobson, 2008).

## Code availability

The R codes used for the analyses for both studies are also available on the Open Science Framework (https://osf.io/q2n7k).

## Declarations

### Open practices statement

The data and analysis files for both studies are available on the Open Science Framework (https://osf.io/q2n7k/). The Israeli Face Database, including face images and demographic details of face identities, can be accessed through instructions detailed the OSF (https://osf.io/q2n7k/). None of the studies were preregistered.
