# Supplementary material for: The Israeli Face Database (IFD): A multi-ethnic database of faces with supporting social norming data
Source: Behav Res Methods. 2025 Jun 13;57(7):197. doi: 10.3758/s13428-025-02723-1 (PMC12165893; doi:10.3758/s13428-025-02723-1)
Supplement: Supplementary file 1 — Supplementary file1 (DOCX 1031 KB) [file 13428_2025_2723_MOESM1_ESM.docx]

**The Israeli Face Database (IFD): A Multi-Ethnic Database of Faces with Supporting Social Norming Data**

**Supplementary Materials**

| Table S1  *The proportion of Correct Expression Recognition, Mean Ratings, and Standard Deviations for Each Facial Expression of the images taken from the Chicago Face Database in Study 1.* | | | | | | | | | | | | | | | | | |
| --- | --- | --- | --- | --- | --- | --- | --- | --- | --- | --- | --- | --- | --- | --- | --- | --- | --- |
| Expression | Valence | |  | Intensity | |  | Clarity | |  | Honesty | |  | Proportion of Correct Recognition | | ≥ 0.667 recognized correctly | Number of Raters |  |
|  | M | SD |  | M | SD |  | M | SD |  | M | SD |  | M | SD |  |  |  |
| Anger | 18.30 | 14.55 |  | 65.09 | 20.11 |  | 62.91 | 22.13 |  | 50.92 | 26.35 |  | 0.73 | 0.32 | 0.60 | 318 |  |
| Happiness, closed mouth | 71.11 | 17.55 |  | 56.54 | 20.82 |  | 60.27 | 22.80 |  | 57.17 | 23.59 |  | 0.78 | 0.32 | 0.80 | 318 |  |
| Happiness, open mouth | 86.90 | 13.21 |  | 79.53 | 16.88 |  | 81.05 | 18.20 |  | 71.49 | 22.28 |  | 0.97 | 0.13 | 1.00 | 318 |  |
| Neutral without mask | 40.31 | 14.23 |  | 59.97 | 21.42 |  | 56.39 | 22.68 |  | 63.19 | 20.29 |  | 0.63 | 0.37 | 0.40 | 318 |  |
| *Note.* Scores represent the means and standard deviations of the mean scores of each face.  We considered both “happiness” and “enjoyment” as correct recognitions of happiness (either with open or closed mouth).  ≥ 0.667 recognized correctly = the proportion of face identities whose expressions were correctly recognized by at least 66.7% of participants. | | | | | | | | | | | | | | | | | |

| \| **Table S2.** \| \| --- \| \| *Comparison Between Ratings of Expression of Faces from Chicago Face Database and Israeli Face Database in Study 1.* \| | | | | | |
| --- | --- | --- | --- | --- | --- | --- | --- |
| Rating criteria and expressions | *t* | df | 95% CI  low | 95% CI high | *p* |
|  |  |  |  |  |  |
| Valence |  |  |  |  |  |
| Anger | 0.95 | 317 | -0.69 | 1.97 | .428 |
| Happiness (closed mouth) | -2.62 | 317 | -4.18 | -0.59 | .026 |
| Happiness (open mouth) | 1.86 | 317 | -0.07 | 2.49 | .105 |
| Neutral | -2.24 | 317 | -3.47 | -0.23 | .058 |
| Clarity |  |  |  |  |  |
| Anger | 1.45 | 317 | -0.63 | 4.16 | .197 |
| Happiness (closed mouth) | -1.83 | 317 | -4.14 | 0.15 | .105 |
| Happiness (open mouth) | 0.30 | 317 | -1.30 | 1.78 | .803 |
| Neutral | -2.08 | 317 | -4.98 | -0.14 | .069 |
| Honesty |  |  |  |  |  |
| Anger | -0.21 | 317 | -2.71 | 2.18 | .831 |
| Happiness (closed mouth) | 0.70 | 317 | -1.44 | 3.06 | .536 |
| Happiness (open mouth) | -1.67 | 317 | -3.78 | 0.31 | .137 |
| Neutral | -2.72 | 317 | -4.59 | -0.74 | .023 |
| Intensity |  |  |  |  |  |
| Anger | 2.81 | 317 | 0.83 | 4.72 | .023 |
| Happiness (closed mouth) | -2.78 | 317 | -4.46 | -0.76 | .023 |
| Happiness (open mouth) | 0.80 | 317 | -0.90 | 2.14 | .499 |
| Neutral | -2.18 | 317 | -4.62 | -0.23 | .060 |
| *Note.* *p* values are corrected for false-rate discovery (FDR).  *df* = degrees of freedom. | | | | | |

**
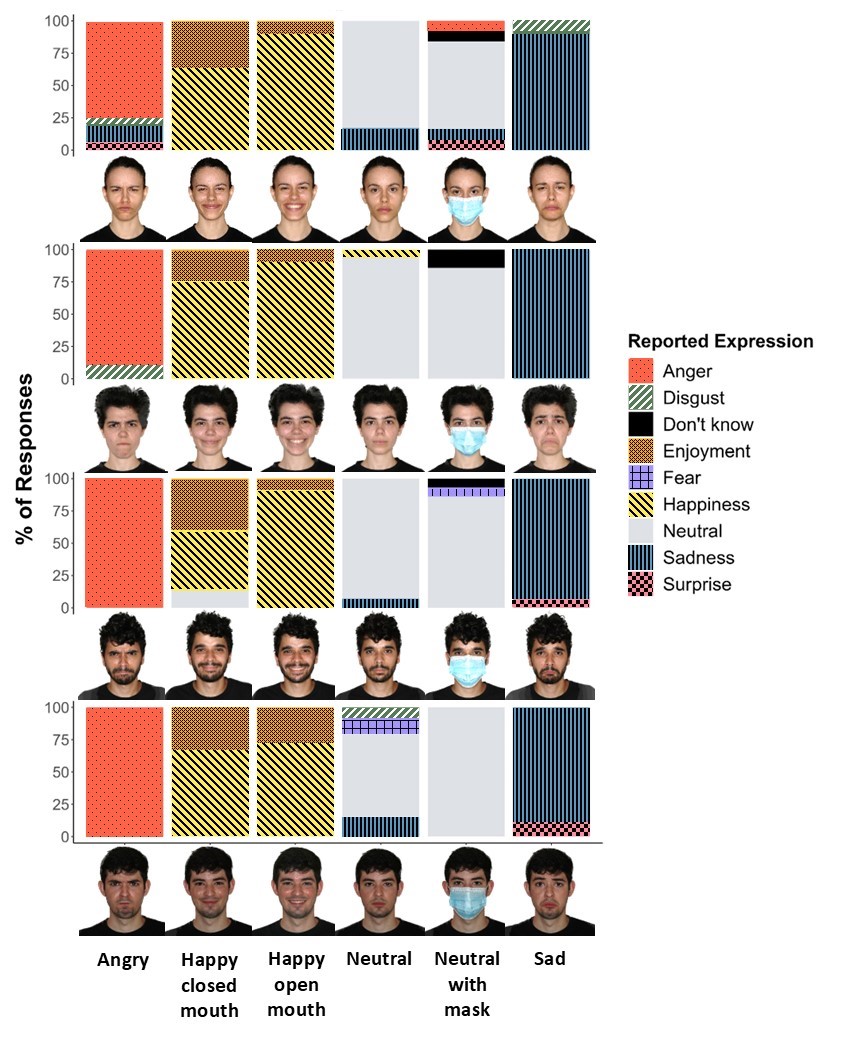
**

**Fig S1.** Examples of IFD face images and distribution of expression recognition responses to six expressions, adapted for impaired vision.

**Additional demographic information regarding rating participants in Study 2**

***Israeli sample***

**Religiosity level.** Among the participants included in the analysis, 57.49% self-identified as non-religious, 21.03% as conservative, 9.06% as moderately religious, 6.29% as religious, 1.12% as ultra-Orthodox, and 5.01% specified other identifications.

**Education level.** Among the participants included in the analysis, 27.10% had completed high school, 37.28% had completed a bachelor's degree (B.A. or B.Sc.), 18.64% had completed a master's degree (M.A., M.B.A., or M.Sc.), 1.95% had completed a Ph.D. or M.D., 12.50% had earned another professional diploma, and 0.75% specified other education levels.

***U.S. sample***

**Religion.** Among the participants included in the analysis, 42.95% self-identified as non-religious, 25.60% as Protestant Christian, 20.77 as Catholic or Orthodox Christian, 2.25% as Muslim, 1.50% as Jewish, 1.08% as Buddhist, Confucian, or Shinto, and 5.85% specified other identifications.

**Education level.** Among the participants included in the analysis, 1.00% had completed Junior high or middle school, 31.69% had completed high school, 39.87% had completed a bachelor's degree (B.A. or B.Sc.), 14.01% had completed a master's degree (M.A., M.B.A., M.Sc.), 3.50% had completed a Ph.D., M.D. or J.D., 0.33% had earned another professional diploma, and 9.59% specified other education levels.

| **Table S3**  *Results of the Comparisons of Social Traits Between the U.S. and Israeli Samples in Study 2.* | | | | | | | | | |
| --- | --- | --- | --- | --- | --- | --- | --- | --- | --- |
| Rating Criterion | Israel | |  | US | |  |  |  |  |
|  | M | SD |  | M | SD |  | *t* | df | *p* |
| Perceived age | 31.51 | 3.04 |  | 31.57 | 3.08 |  | -0.13 | 153.98 | .917 |
| Typicality | 42.99 | 9.47 |  | 32.23 | 11.23 |  | 6.47 | 149.73 | < .001 |
| Aggressiveness | -18.86 | 20.85 |  | -13.11 | 19.17 |  | -1.79 | 152.93 | .153 |
| Attractiveness | -3.03 | 19.83 |  | 5.86 | 19.50 |  | -2.82 | 153.96 | .018 |
| Care | 11.97 | 15.63 |  | 9.05 | 15.19 |  | 1.18 | 153.87 | .331 |
| Dominance | 7.77 | 15.33 |  | 9.10 | 20.37 |  | -0.46 | 143.04 | .775 |
| Emotional stability | 17.33 | 14.64 |  | 10.60 | 15.44 |  | 2.79 | 153.57 | 0.018 |
| Friendliness | 13.97 | 14.93 |  | 2.96 | 18.43 |  | 4.10 | 147.62 | < .001 |
| Meanness | -17.33 | 17.92 |  | -4.46 | 17.41 |  | -4.55 | 153.87 | < .001 |
| Responsibility | 24.16 | 14.50 |  | 25.68 | 14.68 |  | -0.65 | 153.98 | .660 |
| Self-confidence | 18.60 | 14.33 |  | 18.35 | 15.66 |  | 0.10 | 152.80 | .917 |
| Intelligence | 21.06 | 13.95 |  | 23.91 | 9.99 |  | -1.47 | 139.51 | .237 |
| Trustworthiness | 16.72 | 17.10 |  | 12.43 | 13.68 |  | 1.73 | 146.93 | .154 |
| Weirdness | -12.09 | 19.01 |  | -17.22 | 16.90 |  | 1.78 | 151.91 | .153 |
| Femininity (female faces) | 32.39 | 15.69 |  | 41.05 | 14.03 |  | –3.00 | 102.72 | .015 |
| Femininity (male face) | –63.45 | 8.99 |  | –64.07 | 7.42 |  | 0.27 | 46.33 | .889 |
| Masculinity (female faces) | –22.19 | 15.78 |  | –26.04 | 15.60 |  | 1.26 | 103.99 | .314 |
| Masculinity (male faces) | 64.56 | 6.52 |  | 67.82 | 5.79 |  | –1.87 | 47.33 | .153 |
| *Note.* *p* values are corrected for false-rate discovery (FDR).  df = degrees of freedom. | | | | | | | | | |

| **Table S4.**  *Test results of the comparisons of rated social traits to the scale’s middle point.* | | | | | | | |
| --- | --- | --- | --- | --- | --- | --- | --- |
| Rated trait | US | | |  | Israel | | |
|  | *t* | *df* | *P* |  | *t* | *df* | *p* |
| Perceived age | 90.59 | 77 | < .001 |  | 91.54 | 77 | < .001 |
| Typicality | 25.34 | 77 | < .001 |  | 40.09 | 77 | < .001 |
| Aggressiveness | -6.04 | 77 | < .001 |  | -7.99 | 77 | < .001 |
| Attractiveness | 2.66 | 77 | .011 |  | -1.35 | 77 | .182 |
| Care | 5.26 | 77 | < .001 |  | 6.76 | 77 | < .001 |
| Dominance | 3.95 | 77 | < .001 |  | 4.48 | 77 | < .001 |
| Emotional stability | 6.06 | 77 | < .001 |  | 10.45 | 77 | < .001 |
| Friendliness | 1.42 | 77 | .166 |  | 8.27 | 77 | < .001 |
| Meanness | -2.26 | 77 | .029 |  | -8.54 | 77 | < .001 |
| Responsibility | 15.45 | 77 | < .001 |  | 14.71 | 77 | < .001 |
| Self-confidence | 10.35 | 77 | < .001 |  | 11.47 | 77 | < .001 |
| Intelligence | 21.14 | 77 | < .001 |  | 13.33 | 77 | < .001 |
| Trustworthiness | 8.02 | 77 | < .001 |  | 8.64 | 77 | < .001 |
| Weirdness | -9.00 | 77 | < .001 |  | -5.61 | 77 | < .001 |
| *Note.* Scales’ range was -100 to 100, except for perceived age, which was 18-99.  All *p* values are corrected for FDR. | | | | | | | |

| **Table S5.**  *Results of Spearman’s correlation tests between the mean social ratings of face identities given by U.S. and Israeli raters.* | | |
| --- | --- | --- |
| Rated trait | *r* | *P* |
| Perceived age | .95 | < .001 |
| Typicality | .50 | < .001 |
| Aggressiveness | .77 | < .001 |
| Attractiveness | .79 | < .001 |
| Care | .70 | < .001 |
| Dominance | .49 | < .001 |
| Emotional stability | .74 | < .001 |
| Friendliness | .71 | < .001 |
| Meanness | .69 | < .001 |
| Responsibility | .52 | < .001 |
| Self-confidence | .79 | < .001 |
| Intelligence | .61 | < .001 |
| Trustworthiness | .74 | < .001 |
| Weirdness | .66 | < .001 |
| Males' masculinity | .91 | < .001 |
| Males' femininity | .86 | < .001 |
| Females' masculinity | .94 | < .001 |
| Females' femininity | .94 | < .001 |

| **Table S6**  *Results of Levene’s tests comparing heterogeneity of ratings given by U.S. compared to Israeli participants in Study 2.* | | |
| --- | --- | --- |
| Rated trait | *F*(1,154) | *p* |
| Perceived age | 0.07 | .788 |
| Typicality | 3.27 | .073 |
| Aggressiveness | 0.19 | .664 |
| Attractiveness | 0.07 | .792 |
| Care | 0.11 | .743 |
| Dominance | 4.83 | .029 |
| Emotional stability | 0.09 | .767 |
| Friendliness | 3.25 | .073 |
| Meanness | 0.16 | .689 |
| Responsibility | 1.76 | .186 |
| Self-confidence | 1.91 | .169 |
| Intelligence | 7.87 | .006 |
| Trustworthiness | 0.54 | .463 |
| Weirdness | 2.16 | .144 |
| Males' masculinity | 0.39 | .533 |
| Males' femininity | 0.53 | .469 |
| Females' masculinity | 0.14 | .708 |
| Females' femininity | 1.22 | .272 |

**a**
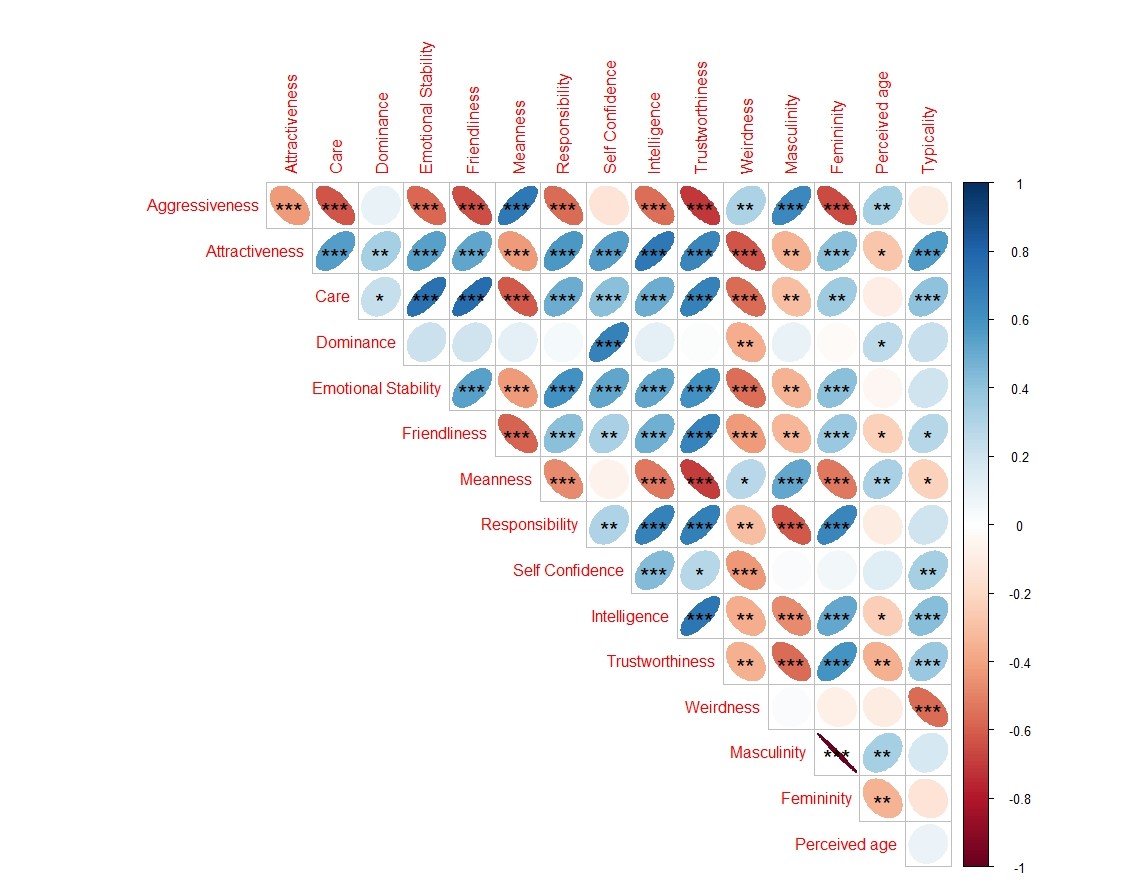


**b**
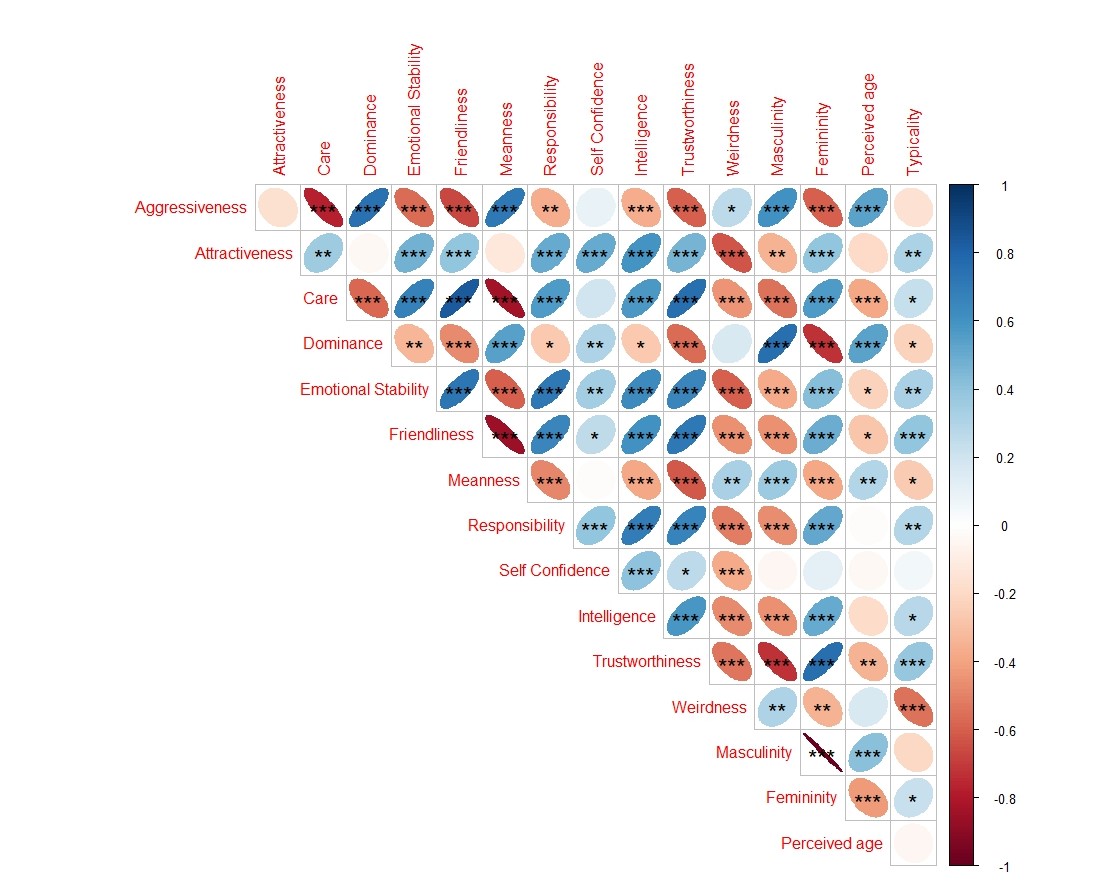


**Fig. S2**

Cross-correlation matrix of average face ratings across multiple traits as rated by (a) the Israeli sample and (b) the U.S. sample. The matrix shows the Spearmen correlation coefficients between each pair of traits. All correlations were computed using the mean ratings for each face across all participants. * = *p* < .05, ** *= p* < .01, *** = *p* < .001.


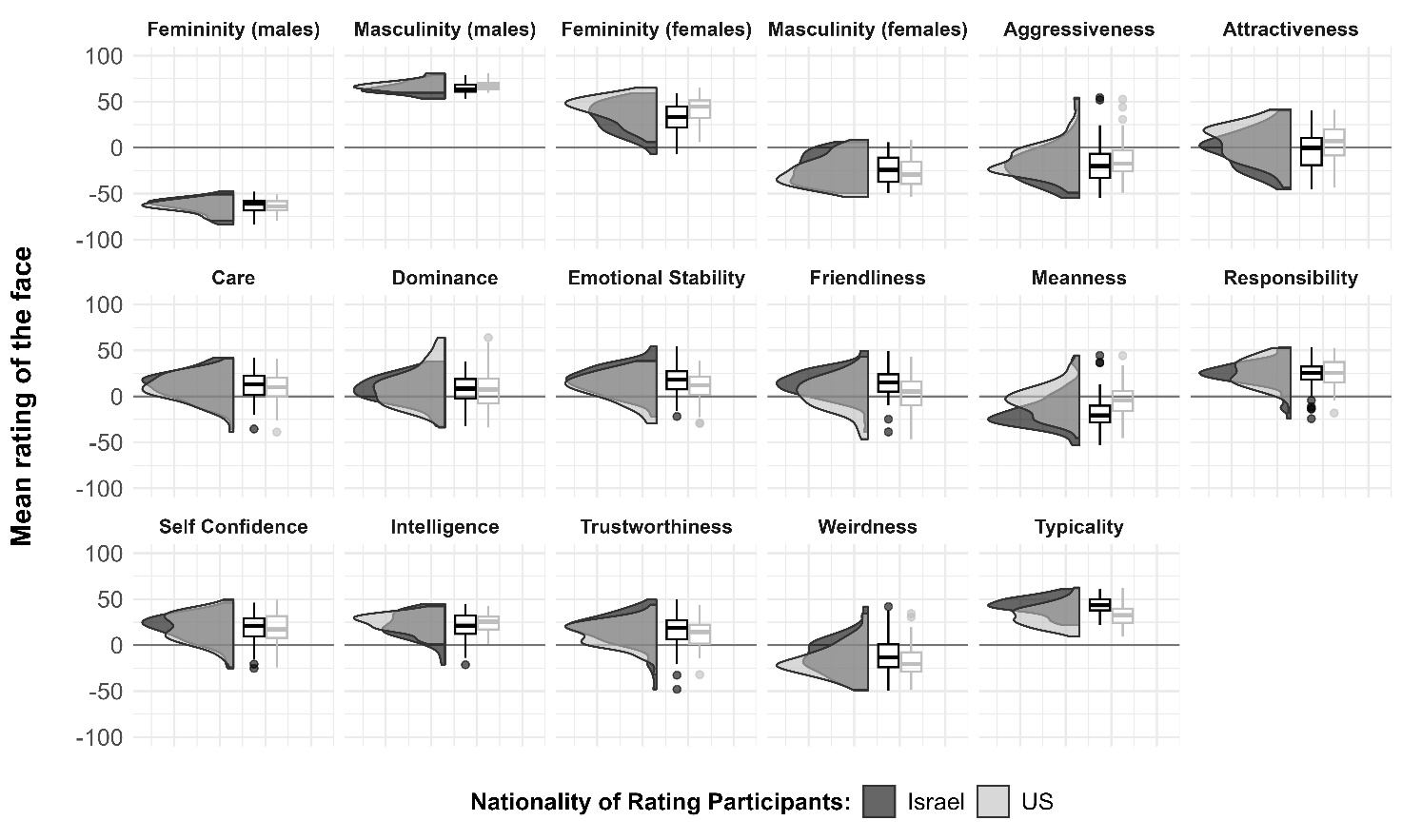


**Fig. S3**

Distributions of social ratings, as rated by U.S. and Israeli participants in Study 2, adapted for impaired vision. * = *p* < .05; *** = *p* < .001.

**
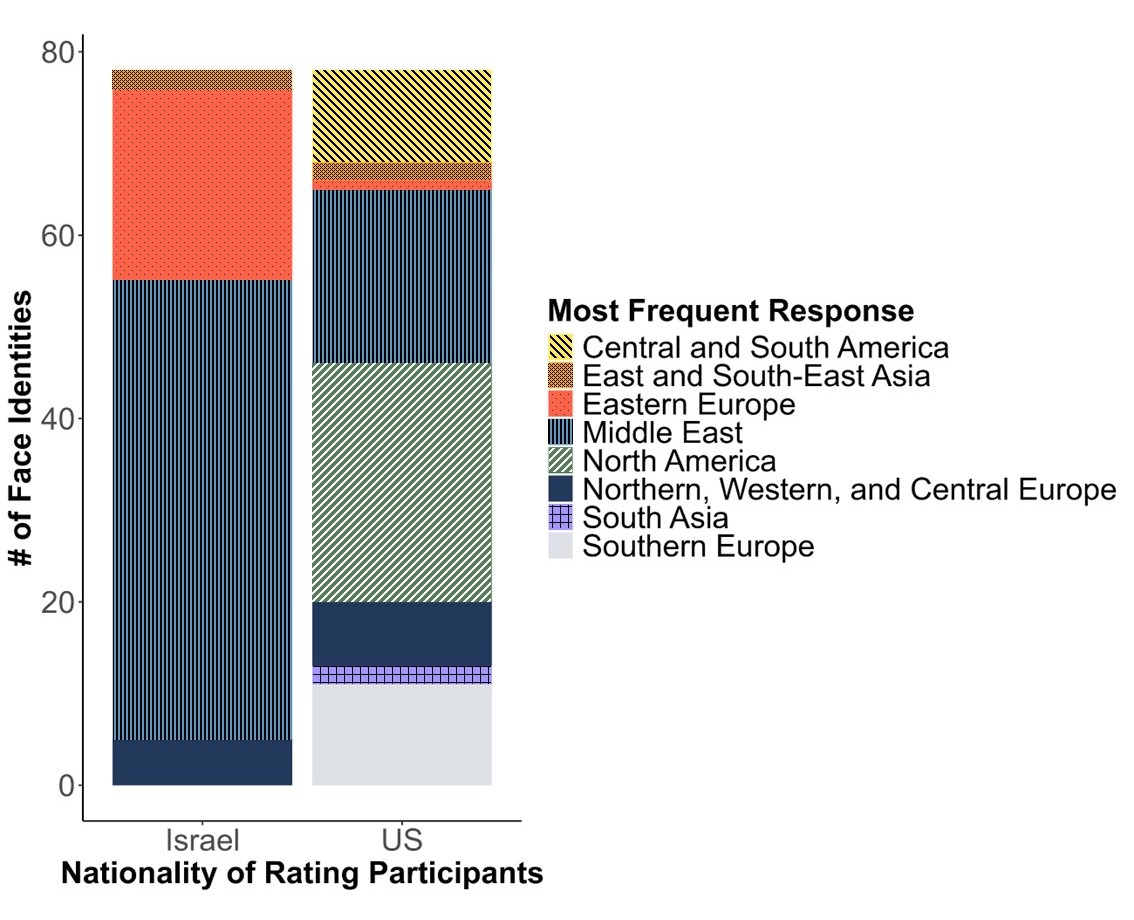
**

**Fig. S4**

Distribution of the most frequent ethnicity categorizations of faces, as categorized by U.S. and Israeli participants, adapted for impaired vision.
